# Transcriptional Analysis of Tissues in Tartary Buckwheat Seedlings Under IAA Stimulation

**DOI:** 10.3390/genes16010030

**Published:** 2024-12-27

**Authors:** Yingying Gao, Jialing Lai, Chenglu Feng, Luyang Li, Qihang Zu, Juan Li, Dengxiang Du

**Affiliations:** 1 School of Life Science and Technology, Wuhan Polytechnic University, Wuhan 430023, China; 2 College of Nursing and Health Management & College of Life Science and Chemistry, Wuhan Donghu University, Wuhan 430212, China; 3 Innovation Institute for Biomedical Material, Wuhan Donghu University, Wuhan 430212, China

**Keywords:** tartary buckwheat, auxin, transcriptome, differentially expressed genes, plant hormone signal transmission

## Abstract

**Background:** *Fagopyrum tataricum*, commonly referred to as tartary buckwheat, is a cultivated medicinal and edible crop renowned for its economic and nutritional significance. Following the publication of the buckwheat genome, research on its functional genomics across various growth environments has gradually begun. Auxin plays a crucial role in many life processes. Analyzing the expression changes in tartary buckwheat after IAA treatment is of great significance for understanding its growth and environmental adaptability. **Methods:** This study investigated the changes in auxin response during the buckwheat seedling stage through high-throughput transcriptome sequencing and the identification and annotation of differentially expressed genes (DEGs) across three treatment stages. **Results:** After IAA treatment, there are 3355 DEGs in leaves and 3974 DEGs in roots identified. These DEGs are significantly enriched in plant hormone signaling, MAPK signaling pathways, phenylpropanoid biosynthesis, and flavonoid biosynthesis pathways. This result suggests a notable correlation between these tissues in buckwheat and their response to IAA, albeit with significant differences in response patterns. Additionally, the identification of tissue-specific expression genes in leaves and other tissues revealed distinct tissue variations. **Conclusions:** Following IAA treatment, an increase in tissue-specific expression genes observed, indicating that IAA significantly regulates the growth of buckwheat tissues. This study also validated certain genes, particularly those in plant hormone signaling pathways, providing a foundational dataset for the further analysis of buckwheat growth and tissue development and laying the groundwork for understanding buckwheat growth and development.

## 1. Introduction

Auxins play a crucial role in cell enlargement, division, and differentiation, as well as in the growth of roots, stems, and leaves, flowering, and fruiting. They are also integral to plants’ responses to external signals [1,2]. Researchers have identified a bioactive growth regulator that promotes seedling growth and have named it auxin. After this substance was purified from plants, it was confirmed to be indole-3-acetic acid (IAA). The effect of auxin on plant growth exhibits a dose–response relationship, with plants maintaining auxin homeostasis in cells and tissues through the regulation of auxin synthesis, binding, degradation, and transport [3,4]. In the isolation of auxin-related plant mutants, researchers uncovered the significant role of auxin in regulating plant growth and development, emphasizing that multiple biological processes—including auxin synthesis, transport, and signal transduction—are also tightly regulated [5,6,7].

Auxin, a crucial plant hormone, plays a pivotal role in regulating various processes of plant growth and development. It is indispensable for the formation of plant nutrient organs, including roots, stems, and leaves [8,9]. Compared to dicotyledonous plants, adventitious roots are more significant for the root structure of grasses. The overexpression of the auxin biosynthesis gene *OsYUCCA1* and polar auxin transport gene *OsPIN1* in rice results in the development of crown roots and lateral roots, as well as an increase in root length [10,11,12]. Conversely, the overexpression of *OsIAA1* in transgenic rice inhibits the formation of both crown and lateral roots. Studies have demonstrated that auxin plays a significant role in root development [13]. Additionally, auxin is essential for stem elongation in both monocotyledonous and dicotyledonous plants. Dwarfism is a common phenotype observed in auxin transport-deficient mutants of maize and rice [14,15]. In maize recessive *Br2* (*brachytic2*) mutants, reduced auxin transport leads to shortened lower stem internodes, and the Br2 gene encodes a class of auxin transport proteins [16,17]. In rice, *NAL1* encodes a specific protein that affects polar auxin transport, and mutations in this gene significantly reduce auxin polar transport, thereby hindering stem elongation [18,19]. There is a defect in the number of leaves in the Arabidopsis pin1 mutant. The leaves of the *NAL7* mutant are notably narrow, but overexpression can widen the leaves. The *NAL7* gene encodes the YUCCA protein involved in auxin biosynthesis. Studies have indicated that auxin also plays a role in leaf development [20,21].

Auxins are known to play a significant role in regulating the reproductive growth process of plants, as well as in the development of flower organs, fruits, and seeds [22,23]. Additionally, auxin is widely recognized as a key regulatory factor in flower organ development [24,25]. Arabidopsis pin1 displays a notable phenotype with the absence of flower formation in the inflorescence. On the other hand, the mutant *ETTIN/ARF3* gene shows significant defects in the pistil structure, such as a reduced ovary size and a displacement of style and stigma tissues [26,27,28,29,30]. This highlights the crucial role of auxin in the development of floral primordia and organ structures. Additionally, auxin is involved in regulating processes like fruit setting, growth, and ripening. Exogenous auxin acting on the ovary can bypass the pollination and fertilization of the fruit, leading to parthenocarpy [31,32]. There is genetic evidence to suggest that *SlIAA9*, *SlARF7*, and Arabidopsis *AtARF8* are involved in fruit setting regulation in tomatoes and inhibit the expression of downstream target genes of auxin before pollination, leading to changes in auxin signal transduction and the formation of parthenocarpy [21,33,34,35]. Research conducted on Arabidopsis has revealed that the temporary rise in auxin levels in the ovary following fertilization triggers auxin signal transduction and controls the expression of downstream target genes, ultimately stimulating fruit growth [36,37]. It has been demonstrated that tomatoes *IAA27* and *ARF10* play a role in the regulation of fruit size and shape [38]. The silencing of the *ARF4* gene, which is strongly expressed in tomato peel, leads to an increase in starch and chlorophyll content accumulation, indicating that auxin is involved in the regulation of chloroplast activity and sugar metabolism in the fruit [31]. In addition, auxin biosynthesis and signal transduction after fertilization serve as the primary driving forces behind seed structure formation [39,40]. The endosperm quality of maize mutants *mn1-1* (miniature1) and dek18 (*defective kernel18*) is significantly diminished, correlating with the downregulation of endosperm-specific genes *ZmYUC1* and *ZmTAR1*, which in turn leads to reduced auxin levels [39,41]. The Arabidopsis mutant of the MADS-box transcription factor *AGL62* is incapable of exporting auxin from the endosperm to the integument, consequently failing to form a seed coat [42,43]. This inability is attributed to the absence of *PGP10* gene expression. Although *PGP10* has not yet been demonstrated to transport auxin, its homologous genes *PGP1*, *PGP19*, and *PGP4* exhibit auxin export functions [44]. Hence, auxin has been established as playing a pivotal role in regulating seed development.

Tartary buckwheat (*Fagopyrum tataricum*) is a multi-purpose plant that integrates nutrition, health care, and medical treatment. It can effectively control diabetes, reduce blood pressure, blood sugar, and blood lipids, eliminate free radicals, enhance immunity, and so on [45,46,47]. Buckwheat has attracted worldwide attention in recent years due to its rich nutritional and medicinal value, as well as its adaptability. It performs well in under-fertilized soil and has high production value and great potential [48]. With the development and application of sequencing technology, transcriptome analysis under different tissue stages and processing conditions has greatly enhanced researchers’ understanding of life activities and biological regulation through increasingly rich expression profiles [49,50]. Through transcriptome analysis, researchers have identified the expression status of buckwheat under different environments such as salt stress [51,52], aluminum stress [53], and drought stress [54]. They have also identified a series of related regulatory pathways and potential candidate genes. In the identification of various tissues during the development of buckwheat, transcriptome analysis was performed on the flowers [55] and developing seeds [56,57], providing a macroscopic analysis of nutrients during seed development. In recent years, studies have shown that IAA regulates the response of plants to adverse environments and the stress resistance of plants by regulating the expression of downstream response genes [58]. There is still a lack of research on the functions of auxin-related regulatory genes, especially the regulatory role in abiotic stress response, as buckwheat is not well studied. At present, there are only a few studies on auxin-related research, such as ARF family analysis and AUX/IAA family analysis [59]. As an important part of plant growth and environmental adaptability, the function of plant hormones including auxin in buckwheat needs further study. This study attempts to understand the molecular mechanism of auxin action in buckwheat, using high-throughput transcriptome analysis. In particular, the expression profiles of multiple families of auxin response in buckwheat growth and development and hormone response were analyzed. This helps to clarify the function of auxin reactive protein and lays a foundation for further research and breeding applications of the buckwheat genome.

## 2. Material and Methods

### 2.1. Plant Material and Cultivation Control

The buckwheat material (*Fagopyrum tataricum*, Jinqiao 2) used in this study was obtained from Taiyuan Normal University (Taiyuan, China). The experiment was carried out in the greenhouse of Wuhan Polytechnic University (Wuhan, China) [60]. Buckwheat seeds were sterilized, rinsed in sterile water, and sown in improved Hoagland solution. The composition of the solution includes calcium nitrate, potassium nitrate, ammonium nitrate, potassium dihydrogen phosphate, magnesium sulfate, and an iron salt solution. This formula has a pH of 6.0, while the iron salt solution, prepared from ferrous sulfate heptahydrate and disodium ethylenediaminetetraacetic acid, has a pH of 5.5. The recipe was followed under standard greenhouse conditions. The culture chamber rooms were set as follows: 14 h day/10 h night cycle, 25/20 °C day/night temperature, 80% relative humidity, and 250 mmolm^−2^ s^−1^ intense luminosity.

### 2.2. Auxin (IAA) Treatments and Material Preparation

When buckwheat plants reach seven leaves, they are treated with IAA under greenhouse conditions. The treatment conditions involve 1 mol/L IAA in Hoagland liquid medium [61]. Following the treatment, fresh leaf and root materials are collected daily, with the untreated materials serving as controls. Thirty samples are taken from each tissue and stored at −80 °C. Tissue RNA is extracted, high-throughput transcriptome analysis is conducted, and gene expression analysis is performed using qRT-PCR.

### 2.3. High Throughput Transcriptome Sequencing

Samples were collected for transcriptome analysis after 1, 2, and 3 days of treatment, using untreated materials as controls. Based on organizational characteristics and processing time, the sample names were designated as Leaf-CK, Leaf-1D, Leaf-2D, Leaf-3D, Root-CK, Root-1D, Root-2D, and Root-3D, with three replicates for each treatment. Total RNA was extracted using a total RNA extraction kit (Sangon, Shanghai, China, SK1321), and genomic DNA was removed by treatment with DNase-I without RNase [62]. According to the manufacturer’s instructions, a library was constructed using the TruSeq Stranded mRNA LTSample Prep Kit (Illumina, San Diego, CA, USA). Sequencing was performed using the Illumina HiSeq platform, and fluorescence image processing, base calling, and quality value calculation were carried out by the Illumina Data Processing Pipeline 1.4 (Illumina^®^, San Diego, CA, USA).

### 2.4. Sequencing Data Analysis and Gene Expression Level Identification

Trimatic was utilized to filter the raw data and eliminate low-quality reads, thereby obtaining clean reads [63]. HISAT2 V2.1.0 was selected for the hierarchical indexing of transcript splicing alignment, to compare transcriptome sequencing reads with reference genomes and identify and classify unique mapped reads and multiple mapped reads [64]. Principal component analysis (PCA) was used to detect the reproducibility and correlation between samples. Cufflinks were used to calculate the transcripts per million mapped reads (TPM) value for each gene, and the adcount for each gene was obtained through htseq counting [65]. Genes were stacked and grouped based on differences in expression levels to identify patterns and relationships within the data.

### 2.5. Comparison of Gene Expression Between qPCR and Transcriptome Analysis

According to the manufacturer’s protocol, total RNA was extracted and genomic DNA was removed using the Trizol method. cDNA was then generated through reverse transcription using M-MLV (TakaRa, Dalian, China). The gene expression of the housekeeping genome protein 3 (GenBank ID: HM628903) was used served as an endogenous control [66]. The expression analysis of ARF genes was based on the study conducted by Liu et al. [59]. The gene-specific primers are detailed in Table 1. The total volume of the qRT-PCR reaction was 20 µL, consisting of 2 µL diluted cDNA, 1 µL each for forward and reverse primers, 10 µL SYBR Premix Ex Taq (Thermo Fisher Scientific, Waltham, MA, USA), and 6 µL ddH_2_O. The qPCR procedure was conducted as follows: The samples were initially heated to 95 °C for 3 min, followed by 30 cycles of denaturation at 95 °C for 15 s, annealing at 60 °C for 30 s, and extension at 72 °C for 20 s. Three technical replicates were analyzed using the 2^−∆∆CT^ method.

### 2.6. Identification and Analysis of Differentially Expressed Genes (DEGs)

Differentially expressed genes (DEGs) were identified using the DESeq package functions estimate Size Factors and nbinomTest. A *p* value of 2 or a fold change <0.5 were set as the thresholds for significant differential expression [67]. The Gene Ontology (GO) enrichment and Kyoto Encyclopedia of Genes and Genomes (KEGG) pathway enrichment analyses of DEGs were performed using R based on the hypergeometric distribution. Data from http://www.geneontology.org/ and http://www.genome.jp/kegg/ (accessed on 30 September 2024) were utilized for the analysis [68].

### 2.7. Identification and Expession Analysis of Auxin Signaling Pathway Genes

From the Pfam family database (http://pfam.xfam.org/search, accessed on 30 September 2024), Hidden Markov Model (HMM) profiles were obtained for ARF (PF06507), Aux/IAA domains (PF02519), GH3 (PF03321), SAUR (PF0259), AUX1 (PF01490), and TIR1 (PF12937). These profiles were used to identify genes belonging to each family member in the Tartary Buckwheat genome (http://www.mbkbase.org/Pinku1/, accessed on 30 September 2024) [69]. Based on annotation files and sequence alignment, duplicate genes were removed, and the remaining genes were identified as auxin signaling pathway genes. Transcriptome data were then utilized for gene expression analysis across different tissues and under hormone treatment, aiming to identify significantly differentially expressed genes.

### 2.8. Data Availability Statement

The experiments were performed with three biological replicates, and statistical significance was determined using a Two-way ANOVA and Tukey’s test. Statistical significance was denoted as *** *p* ≤ 0.001, ** *p* ≤ 0.01, and * *p* ≤ 0.05.

## 3. Results

### 3.1. Statistics of Expressed Genes Through High-Throughput Transcriptome Analysis

An overview of the reads derived from the tissues was listed in Table 2, totaling 163.62 GB of clean data. The average size of each sample is 6.81 GB, with a Q30 base distribution range of 89.64~90.54% and an average GC content of 45.61%. All clean reads were successfully mapped to the reference genome data, achieving a genome alignment rate of 81.93–99.96% for each sample. Following the removal of low-quality tags, between 18,874,534 (Leaf-CK-3) and 19,975,996 (Root-3D-3), clean tags were successfully mapped to the reference genome. Among the total clean reads from the 24 libraries, approximately 79.80% and 97.94% were unique when compared to the buckwheat genome, whereas 1.06% and 3.70% were multiply mapped.

**Figure 1:**
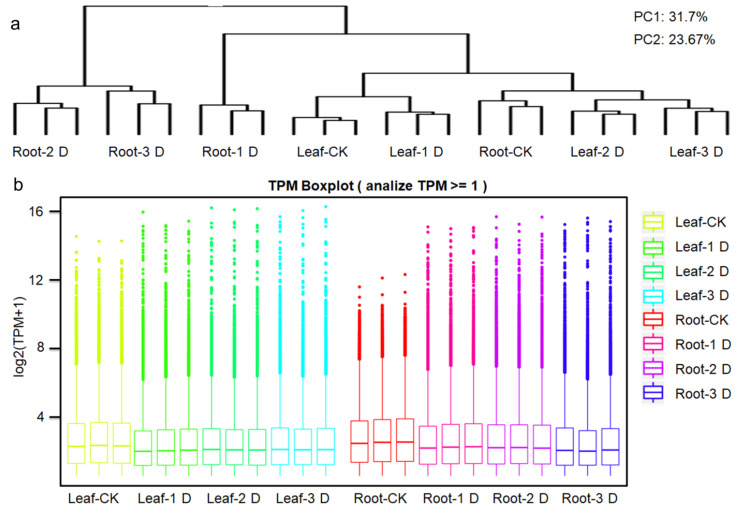
Statistics of transcriptome data using high-throughput deep sequencing technology. (**a**) Relationship between samples in three biological replicates and summary of expressed genes in different treatments. Additionally, the comparison of gene expression levels across treatments will be analyzed. (**b**) The boxplot of the expressions of 24 samples will be presented to visually represent the distribution of gene expression data. The expression density of genes in twelve samples was represented by Transcripts Per Million mapped reads (TPM). The abscissa represented the sample name, while the ordinate represented log2 (TPM + 1). The box chart for each region is divided into five statistics (from top to bottom: the maximum, the upper quartile, the median, the lower quartile, and the minimum).

PC1 and PC2 accounted for 31.70% and 23.67% of the transcriptome variation in 24 samples, respectively. The PCA results for the 24 samples demonstrated significant separation among different tissue groups. Additionally, the repeated experiments for each treatment clustered together (Figure 1a). Across the eight groups of materials in this study, all three experimental replicates exhibited a correlation coefficient exceeding 0.96, indicating that each replicate was suitable for subsequent analysis. The transcript abundance (TPM) of each gene was estimated by the number of uniquely mapped reads overlapping with the exon region, based on the transcript abundance per million mapped reads (TPM). Genes that were detected twice in three replicates and had an average TPM expression level exceeding 0.5 were selected as expressed genes (Figure 1b). A total of 29,757 expressed genes were identified, with 24,529 to 27,813 expressed genes identified in different materials (Figure 1). In the eight groups of materials, 27,227 (Leaf-CK), 26,222 (Leaf-1D), 26,548 (Leaf-2D), 26,432 (Leaf-3D), 26,892 (Root-CK), 27,073 (Root-1D), 26,919 (Root-2D), and 27,468 (Root-3D) expressed genes were detected, respectively.

### 3.2. Comparison of Gene Expression Between qRT-PCR and Transcriptome Analysis

In order to compare gene expression between qRT-PCR and transcriptome analysis, we conducted a comprehensive analysis of the data obtained from both methods. This allowed us to assess the consistency and reliability of the results generated by each technique. By examining the correlation between the expression levels of specific genes as measured by qRT-PCR and transcriptome analysis, we were able to determine the degree of agreement between the two methods. Additionally, we performed statistical analyses to identify any potential discrepancies or biases that may have influenced the results. Overall, our findings suggest that while there may be some differences between the two techniques, they generally provide complementary information that can be used to enhance our understanding of gene expression patterns.

**Figure 2:**
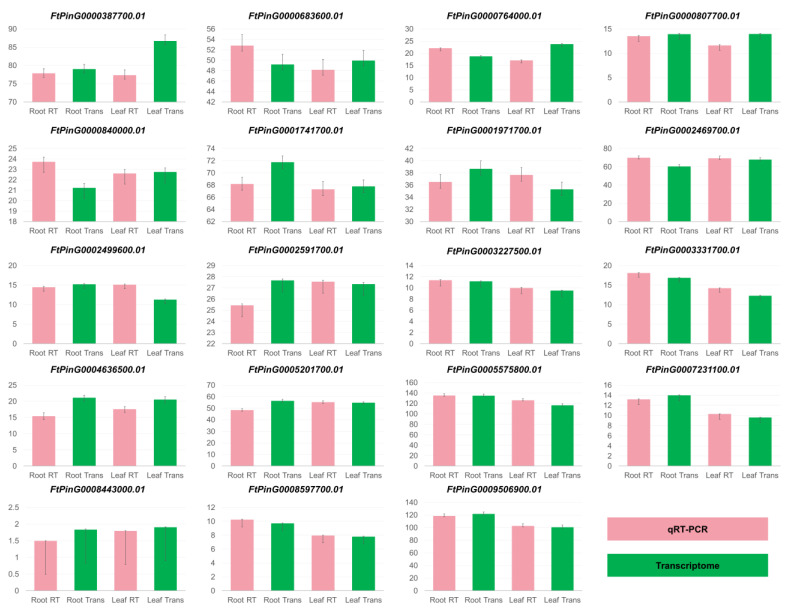
Expression profiles of genes of ARF based on the transcriptome analysis and qRT-PCR. Green represented gene expression levels of transcriptome analysis. Pink represented gene expression levels of qRT-PCR analysis.

To validate the expression profile obtained through transcriptome analysis, real-time PCR was conducted. The 19 genes of the ARF gene family exhibited significantly distinct expression patterns across the four stages (Figure 2). Overall, the concordance rate between RNA-seq and qRT-PCR stood at 90%, indicating the high accuracy of RNA-seq and the reliability of the identified pathways and candidate genes. The absence of detected gene expression levels in some samples suggests that DEGs were not expressed at this stage. qPCR testing demonstrated a high degree of consistency between the transcriptome data from this study and the results obtained through real-time quantitative PCR analysis. Therefore, the gene expression levels determined through sequencing in this study can serve as a reliable basis for identifying the expression levels of additional genes.

### 3.3. Identification of Differentially Expressed Genes (DEGs) in Leaf

**Figure 3:**
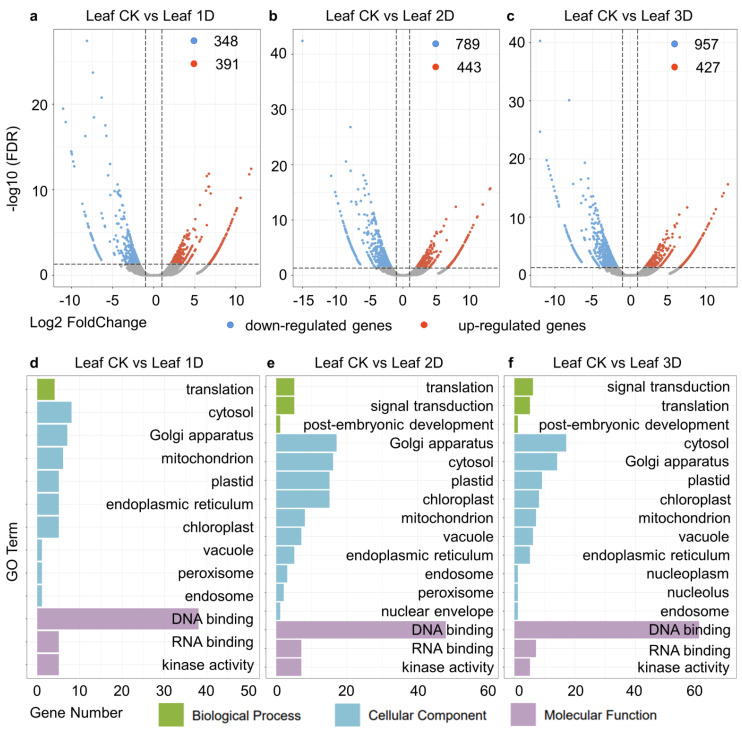
Identification and functional analysis of DEGs in leaves treated with IAA. (**a**–**c**) Volcano plot of DEGs between IAA treatment and control. Red represented up-regulated genes, blue represented down-regulated genes, and gray represented genes with no significant expression changes. The horizontal axis represented the log2 FoldChange value, and the vertical axis represented the −log10 (FDR) value. (**d**–**f**) GO enrichment analysis of DEGs between IAA treatment and control. The horizontal axis represented RichFactor, and the vertical axis represented GO Term.

Using the leaves before hormone treatment as a control, we detected differentially expressed genes in the leaves at different treatment stages. In this study, after one day of IAA treatment, 391 genes were upregulated and 348 genes were downregulated in the leaves (Figure 3a). The DEGs were enriched in 14 Gene Ontology (GO) categories, which are divided into biological processes, cellular components, and molecular functions. Among them, DNA binding, cytoplasm, and Golgi apparatus rank as the top three GO terms (Figure 3d). Between the second day of treatment and the control group (Leaf-CK and Leaf-2D), 443 up-regulated genes and 789 downregulated genes obtained (Figure 3b). There are a total of 16 GO terms classified into biological processes, cellular components, and molecular functional categories. In addition, DNA binding, Golgi apparatus, cytoplasm, plastid, and chloroplast are the top five GO terms with the highest number of genes, containing 48, 17, 16, 15, and 15 genes, respectively (Figure 3e). In the third stage (Leaf-CK vs. Leaf-3D), 427 genes were up-regulated and 957 genes were down-regulated in the leaves after IAA treatment (Figure 3c). Among them, kinase activity (five genes), DNA binding (61 genes), and RNA binding (seven genes) belong to molecular functions. During the cellular composition process, 10 GO terms were identified, with the cytoplasm (17), Golgi apparatus (14), plastids (nine), chloroplasts (eight), and mitochondria (seven) containing the most genes. Four GO terms related to biological processes, namely translation, signal transduction, and post-embryonic development, were identified (Figure 3f).

**Figure 4:**
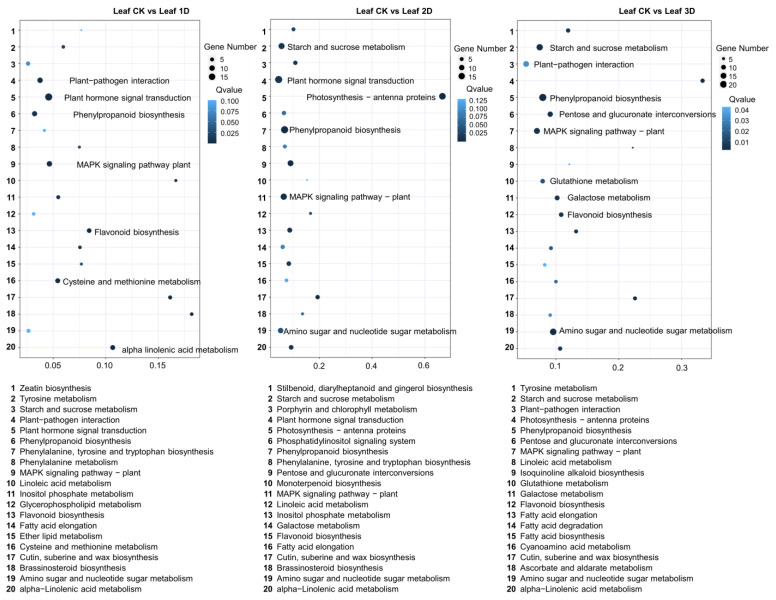
KEGG enrichment analysis of DEGs between IAA treatment and control groups. The horizontal axis represented RichFactor, and the vertical axis represented path. The larger the diameter of the bubble, the richer the genes, while the darker the color of the bubble, the higher the *p*-value.

Using the leaves before hormone treatment as a control, we detected differentially expressed genes in the leaves at different treatment stages. During the Kyoto Encyclopedia of Genes and Genomes (KEGG) pathway analysis, we identified 80 pathways (Figure 4). Figure displays the top 20 pathways, among which plant hormone signaling pathway (19), MAPK signaling pathway plant (10), plant–pathogen interaction (10), phenylpropanoid biosynthesis (9), alpha linolenic acid metabolism (8), cysteine and methionine metabolism (8), and flavonoid biosynthesis (7) contain the most genes. Between the second day of treatment and the control group (Leaf-CK and Leaf-2D), 91 KEGG pathways were identified, and the top 20 pathways were identified, including plant hormone signal transduction (19), phenylpropanoid biosynthesis (19), photosynthetic antenna proteins (16), MAPK signaling pathway in plants (14), starch and sucrose metabolism (13), and amino sugar and nucleotide sugar metabolism (12). During the third stage, a total of 94 KEGG pathways were identified. The top 20 pathways including phenylpropane biosynthesis (22), amino sugar and nucleotide sugar metabolism (18), plant hormone signal transduction (17), starch and sucrose metabolism (17), MAPK signaling pathway in plants (15), plant-pathogen interaction (14), pentose and glucuronate interconversion (12), galactose metabolism (10), flavonoid biosynthesis (9), and glutathione metabolism (9), which have the most enriched genes. Throughout these three stages, four pathways were consistently identified, namely plant hormone signaling transduction, MAPK signaling pathway in plants, phenylpropanoid biosynthesis, and flavonoid biosynthesis.

**Figure 5:**
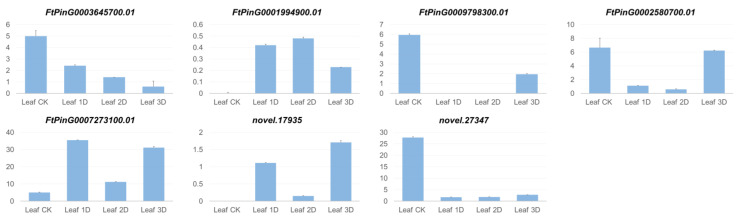
Genes with significant changes in expression present in pathway plant hormone signal transmission in leaves.

Plant hormone signal transmission was significantly enriched in genes in the three periods in leaves after the IAA treatment. Significant expression changes were detected for 19 genes in Leaf-CK vs. Leaf-1D, 19 genes in Leaf-CK vs. Leaf-2D, and 17 genes in Leaf-CK vs. Leaf-3D, respectively. Among these, five genes, *FtPinG0006457000* (protein ethylene insensitive), *FtPinG0001994900* (transcription factor perianthia), *FtPinG0009798300* (xyloglucan endotransglucosylase), novel.27347 (jasmonoyl–L-amino acid synthetase), and novel.17935 (ethylene receptor 2), showed significant expression changes in all three treatment periods. Two genes, *FtPinG0002580700* (jasmonoyl–L-amino acid synthetase) and *FtPinG0007273100* (auxin-responsive protein IAA26), exhibited changes in expression levels during at least two periods. Figure 5 displays these genes, which demonstrated significant expression alterations across multiple treatment stages. The figure indicates significant changes in gene expression on the first day of treatment. Four genes (*FtPinG0006457000*, *FtPinG0009798300*, *novel.27347*, *FtPinG0002580700*) were downregulated, while three genes (*FtPinG0001994900*, *novel.17935*, *FtPinG0007273100*) were upregulated. On the second day, three genes (*FtPinG0006457000*, *FtPinG0001994900*, *FtPinG0002580700*) maintained their trend from the first day, but the changes were not as pronounced. The trend of change in two genes (*novel.17935*, *FtPinG0007273100*) was significantly downregulated, while the expression of two genes (*FtPinG0009798300*, *novel.27347*) remained unchanged compared to the first day of treatment. On the third day of treatment, most genes showed significant changes, with gene *FtPinG0006457000* maintaining a consistent trend of change. The expression of gene FtPinG0002580700 was upregulated and restored to the same level as the untreated material. There were also *FtPinG0001994900* genes with expression recovery, but there were still significant changes compared to the control. Genes *FtPinG0009798300*, *FtPinG0007273100* and *novel.17935* showed significant upregulation compared to the two strong treatment periods.

### 3.4. Identification of Differentially Expressed Genes (DEGs) in Root

**Figure 6:**
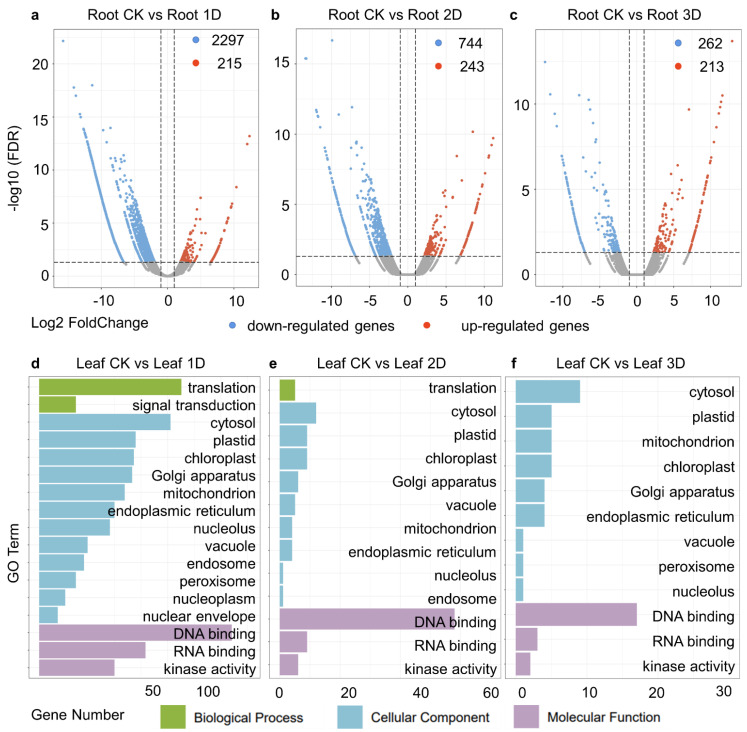
Identification and functional analysis of DEGs in roots treated with IAA. (**a**–**c**) Volcano plot of DEGs between IAA treatment and control. Red represented up-regulated genes, blue represented down-regulated genes, and gray represented genes with no significant expression changes. The horizontal axis represented the log2FoldChange value, and the vertical axis represented the −log10 (FDR) value. (**d**–**f**) GO enrichment analysis of DEGs between IAA treatment and control. The horizontal axis represented RichFactor, and the vertical axis represented GO Term. The larger the diameter of the bubble, the richer the genes, while the darker the color of the bubble, the higher the *p*-value.

After one day of IAA treatment in the roots, 215 genes were upregulated and 2297 genes were downregulated in the leaves (Figure 6a). DEGs were enriched in 18 gene ontology (GO) categories, including biological processes, cellular components, and molecular functions. Among them, translation, DNA binding, cytoplasm, RNA binding, and Golgi apparatus were the top five GOs (Figure 6d). On the second day of treatment, compared to the control group (Roo-CK vs. Root-2D), 243 upregulated genes and 744 downregulated genes were identified (Figure 6b). There were 14 GO categories divided into biological processes, cellular components, and molecular functions. In addition, DNA binding, cytoplasm, plastid, chloroplast, and RNA binding were the top five GOs with the highest number of genes, containing 58, 12, nine, nine, and nine genes, respectively (Figure 6e). In the third stage (Root-CK vs. Root-3D), 213 genes were upregulated and 262 genes were downregulated in the leaves after IAA treatment (Figure 6c). Among them, kinase activity (two genes), DNA binding (17 genes), and RNA binding (three genes) were identified as molecular functions. During the process of cell composition, nine GO terms were identified: nucleolus (1), mitochondria (5), vacuoles (1), peroxisomes (1), endoplasmic reticulum (4), Golgi apparatus (4), cytoplasm (9), plastids (5), and chloroplasts (5) (Figure 6f).

**Figure 7:**
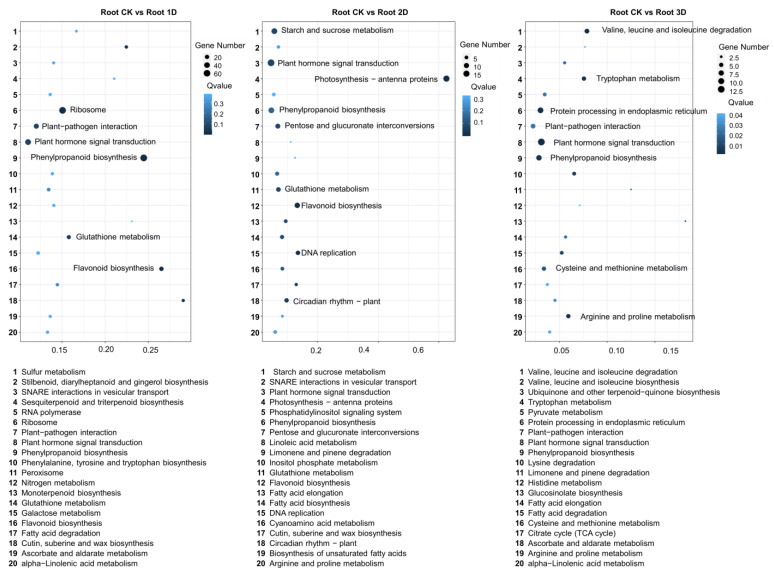
KEGG enrichment analysis of DEGs between IAA treatment and control groups. The horizontal axis represented RichFactor, and the vertical axis represented path. The larger the diameter of the bubble, the richer the genes, while the darker the color of the bubble, the higher the *p*-value.

After one day of IAA treatment in the roots, the KEGG pathway analysis of DEGS obtained 110 pathways identified. Figure 7 displays the top 20 pathways, among which phenylpropanoid biosynthesis (68), ribosomes (66), plant hormone signaling transduction (46), plant-pathogen interactions (32), flavonoid biosynthesis (22), starch and sucrose metabolism (22), and glutathione metabolism (18) contained the most genes. On the second day of treatment, 85 KEGG pathways were identified, including plant hormone signal transduction (19), photosynthetic antenna proteins (15), starch and sucrose metabolism (13), phenylpropanoid biosynthesis (13), flavonoid biosynthesis (11), pentose and glucuronate interconversion (9), glutathione metabolism (8), MAPK signaling pathway in plants (7), DNA replication (7), circadian rhythm in plants (6), and ubiquitin-mediated proteolysis (7). In the third stage, a total of 64 KEGG pathways were identified; the top 20 pathways are shown in Figure 7. Plant hormone signal transduction (13), endoplasmic reticulum protein processing (9), phenylpropanoid biosynthesis (8), valine, leucine, and isoleucine degradation (6), plant-pathogen interaction (6), and tryptophan metabolism (5) enriched the most genes. During these three stages, four pathways were consistently identified, including plant hormone signaling transduction, MAPK signaling pathway, and phenylpropanoid biosynthesis.

**Figure 8:**
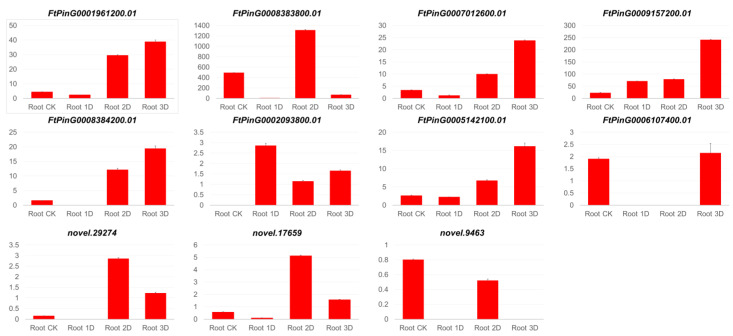
Genes with significant changes in expression present in pathway plant hormone signal transmission in roots.

In roots, 46 genes (Root-CK vs. Root-1D), 18 genes (Root-CK vs. Root-2D) and 13 genes (Root-CK vs. Root-3D) were genes obtained revealing significant expression changes (Figure 8). Among these, the gene *FtPinG0001961200* (auxin-induced protein 22D-like) showed significant expression changes in all three treatment periods. Additionally, 10 genes, *FtPinG0008383800* (pathogenesis-related protein 1A-like), *FtPinG0007012600* (auxin-induced protein 22B-like), *FtPinG0009157200* (hypothetical protein), *FtPinG0008384200* (hypothetical protein), *FtPinG0002093800* (transcription factor TGA3-like), *FtPinG0005142100* (auxin-induced protein AUX22), *FtPinG0006107400* (two-component response regulator ORR9-like), *novel.29274* (putative SAUR-like auxin-responsive protein), *novel.17659* (auxin-induced protein 22D-like), and *novel.9463* (indole-3-acetic acid-amido synthetase GH3.6-like), showed expression changes in at least two periods. Similar changes were detected in the root, with most gene treatments showing significant expression changes on the first day. Specifically, nine genes exhibited decreased expression, while two genes showed an upward trend. Among the downregulated genes, seven genes showed significant changes, and the downregulated expression trend of the two genes was not significant, while the upregulated genes showed a significant change trend (Figure 6). On the second day of treatment, nine genes showed significant expression changes, of which only two genes maintained the same trend of change on the third day of treatment. This phenomenon clearly demonstrates that the genes in the roots undergo more complex changes compared to the leaves. After IAA treatment, a higher number of genes are detected in the roots, which may be attributed to the direct contact and absorption of IAA by the roots.

Furthermore, during the same period of treatment, differentially expressed genes for the plant hormone signal transduction pathway were detected in both leaves and roots, exhibiting a certain overlap. Seven genes were detected on the first day of treatment and were differentially expressed in both leaves and roots. These genes were *novel.15415*, *FtPinG0008047100*, *novel.15414*, *novel.29618*, *FtPinG0009798300*, *FtPinG0008383800*, and *FtPinG0003482900*. This indicates that similar responses were produced in the early stages of treatment in both tissues. On the second and third day of treatment, three overlap genes (*FtPinG0008384200*, *FtPinG0006457000*, *novel.27347*) and one overlap gene (*FtPinG0004642000*) were detected, indicating that different regulatory responses were generated in different tissues during the later stage of treatment.

### 3.5. Specifically Expressed Genes in Leaves and Roots, as Well as DEGs in Various Tissues

**Figure 9:**
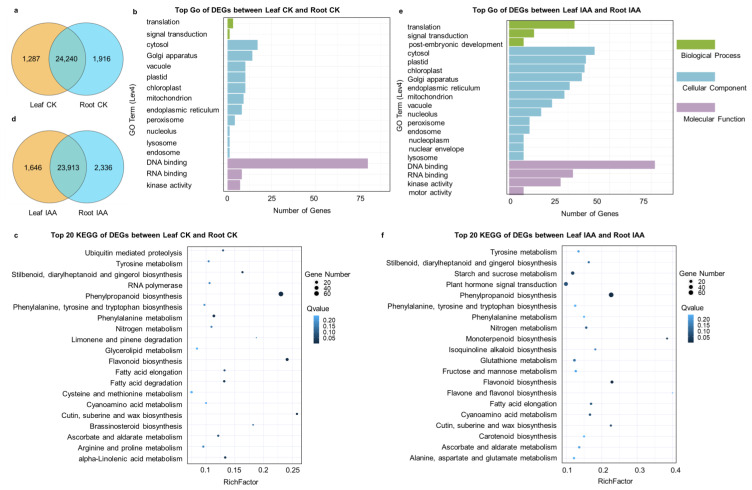
Expression gene analysis in leaves and roots before and after IAA treatment. (**a**) The differential expression of genes in leaves and roots shown in the Venn diagram before IAA treatment. (**b**) The differential gene GO enriched between leaves and roots before IAA treatment. The horizontal axis represented RichFactor, and the vertical axis represented GO Term. A larger bubble diameter indicated more enriched genes, while a darker bubble color indicated a higher *p*-value. (**c**) The differentially expressed gene KEGG enriched between leaves and roots before IAA treatment. The horizontal axis represented RichFactor, and the vertical axis represented pathway. A larger bubble diameter indicated more enriched genes, while a darker bubble color indicated a higher *p*-value. (**d**) The differential expression of genes in leaves and roots after IAA treatment depicted in the Venn diagram. (**e**) The differential gene GO was enriched between leaves and roots after IAA treatment. The horizontal axis represented RichFactor, and the vertical axis represented GO Term. A larger bubble diameter indicated more enriched genes, while a darker bubble color indicated a higher *p*-value. (**f**) The differential gene KEGG enriched between leaves and roots after IAA treatment. The horizontal axis represented RichFactor, and the vertical axis represented pathway. A larger bubble diameter indicated more enriched genes, while a darker bubble color indicated a higher *p*-value.

Comparing the expressed genes in the leaves and roots of tartary buckwheat during the seedling stage, we found that 24,240 genes were detected in both tissues. Additionally, 1287 genes were specifically expressed in the leaves and 191 genes were specifically expressed in the roots (Figure 9a). GO analysis of tissue characteristic expression genes revealed that they were enriched in one class of biological process, cellular component, and molecular function (Figure 9b). The significantly enriched classes include DNA binding, RNA binding, plastid, golgi apparatus, and cytosol. Further KEGG analysis enriched these genes in 63 pathways, among which the most significantly enriched ones included 20 KEGGs such as phenylpropanoid biosynthesis, phase and glucose introversion, flatter biosynthesis, fatty acid degradation, and alpha linolenic acid metabolism (Figure 9c). After IAA treatment, more specifically expressed genes were detected in both leaves and roots, with 1646 (leaves) and 2336 (roots) genes, indicating specific responses in different tissues (Figure 9d). After GO enrichment, classes similar to those before treatment were found, mainly due to differences in the number of enriched genes (Figure 9e). The top 20 KEGGs were enriched in 74 KEGG pathways, as shown in Figure 9f. Most of the KEGGs were similar to before treatment, and significant enrichment was detected in pathways such as phenylpropanoid biosynthesis, phase and glucose introversion, flatter biosynthesis, fat acid degradation, and alpha linolenic acid metabolism. Consistent performance was detected before treatment, suggesting that these pathways are primarily influenced by tissue differences. Significant enrichment was observed in pathways such as plant hormone signal transduction, start-up, and crossover metabolism, indicating the presence of IAA responsive genes in various tissues.

**Figure 10:**
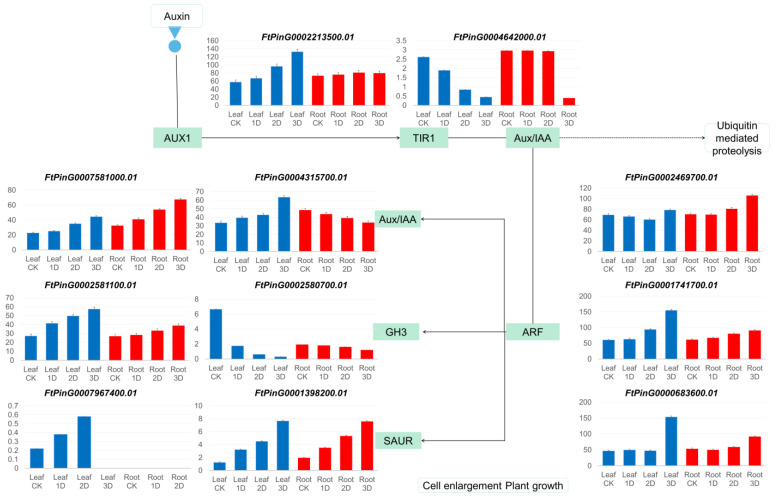
Genes with significant changes in expression presented in pathway plant hormone signal transmission.

Eleven genes from the auxin-responsive family showed significant changes in expression levels (Figure 10). This indicates that these genes involved in hormone response signals significantly regulated in leaves after IAA treatment, providing a basis for buckwheat to adapt to high concentrations of IAA treatment. In addition, four genes, *FtPinG0009798300*, *FtPinG0006457000*, *FtPinG0001994900*, and *novel.27347*, obtained at the three stages. Genes *FtPinG0002580700*, *FtPinG0007273100*, *FtPinG0002889200*, *FtPinG0004641200.01*, and *novel.15911* obtained in at least two stages. The analysis of these genes provides valuable insights into the genetic mechanisms underlying the developmental processes studied.

### 3.6. Identification and Expression Analysis of Auxin Signaling Pathway Genes

**Figure 11:**
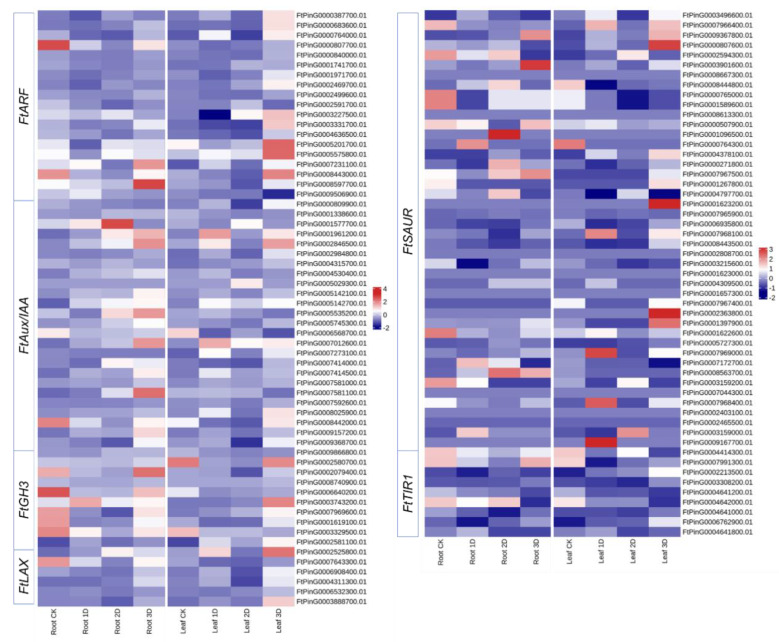
The gene expression levels of the *FtARFs*, *FtAux/IAAs*, *FtGH3s*, *FtSAURs*, *FtLAXs*, and *FtTIR1s* genes at four stages with IAA treatment are represented by heat maps. In the heat maps, the expression levels are indicated by darker red colors, as shown in the top right legend. *FtARFs* represents genes belonging to the ARF family in buckwheat, *FtAux/IAAs* represents genes belonging to the Aux/IAA family, *FtGH3s* represents genes belonging to the GH3 family, *FtSAURs* represents genes belonging to the SAUR family, *FtLAXs* represents genes belonging to the LAX family, and *FtTIR1s* represents genes belonging to the TIR1 family.

Transcriptome analysis used to determine the expression profiles of hormone signaling pathway family genes, as shown in Figure 11. The results indicate that the FtARFs, FtAux/IAA, and FtLAXs families exhibit high levels of gene expression. Upon IAA treatment, certain genes within the FtGH3s and FtTIR1s families exhibited enhanced expression, whereas others demonstrated reduced expression levels. The expression of most FtSAUR family genes remains relatively low. Within each family, distinct expression changes noted before and after hormone treatment. The majority of genes demonstrated upregulation, although some exhibited a decreasing trend or no significant changes. The expression alterations of hormone response family genes and plant hormone signaling pathway genes illustrate the regulation of hormone levels in buckwheat following IAA hormone treatment, thereby facilitating adaptation to IAA stimulation (Figure 11). In the ARF family, the expression of six genes gradually increased over time. Specifically, six genes exhibited significant upregulation during the early stages of treatment but later reverted to downregulation. Additionally, four genes demonstrated a decreasing trend during treatment, whereas two genes maintained low expression levels throughout the entire process. The Aux/IAA family comprises eight highly expressed genes, nine moderately expressed genes, and one low-expressed gene. Following hormone treatment, 11 genes were upregulated, while seven genes were downregulated. Within the GH3 family of genes, five genes exhibited significant upregulation and continued to increase as treatment duration extended. One gene demonstrated slight upregulation, whereas three genes exhibited no significant changes. Among the 54 SAUR genes, 13 genes consistently showed upregulation. Following IAA treatment, notable changes in expression were observed in 20 genes (mildly upregulated), nine genes (slightly downregulated), and 11 genes (no significant changes). In the LAX family, hormone treatment led to the significant upregulation of four genes in both leaves and roots, albeit with fluctuations towards downregulation on the second day of treatment. Within the TIR1 family, one gene exhibited no change in expression, three genes showed no significant change, and four genes were significantly upregulated.

## 4. Discussion

Plant hormones can be used as key endogenous factors that mediate plant stress response. Auxin signaling is a key factor in many plant biological processes, such as growth, development for most organogenesis, and response to a variety of environmental changes [70,71,72]. Phytohormones are the integration center for plants to respond to environmental stimuli. They play an important role in plant defense response. Through the complex signal network and intricate cross-dialogue between different hormone signal pathways, plants can realize the fine regulation of stress response. The adjustment of plant growth response and the improvement of stress resistance level are very important for their survival [8,73].

When plants are grown under IAA conditions, their adaptive mechanisms are adjusted to maintain the stability of life processes by regulating several major clusters, as auxin plays a significant role in many major development processes and cell state maintenance [74,75]. Hormone-induced expression and hormone-responsive genes have been widely isolated from Tartary buckwheat, based on high-throughput sequencing analysis. In this study, we investigated the genetic diversity of auxin response in buckwheat during the seeding stage. All 21,422 identified genes were expressed with varying expression patterns during different stages after IAA treatment. We identified a total of 3355 (1261 up-regulated and 2094 down-regulated) genes to be differentially expressed between the samples after IAA treatment and the control. All the DEGs were divided into the biological process, cellular component, and molecular function categories. In particular, these genes were significantly enriched in the pathways of phylopropanoid biosynthesis, amino sugar and nucleoside sugar metabolism, starch and sugar metabolism, plant hormone signal transmission, MAPK signaling pathway–plant, plant–pathway interaction, pentose and gluconate interconversion, galactose metabolism, flavonoid biosynthesis, glutathione metabolism, and cystaine and methionine metabolism.

Gene expression differences among individuals of the same species are divided into transcriptional variation and translation variation. The differences in gene expression can be allele-specific and local to the regulated gene, tissue-specific expression, and conditionally induced expression [76]. Transcriptome profiling has been used to characterize gene expression variation. These differences in expression include many quantitative differences [77]. There are examples of developmental transcriptome atlases for many plant species [78,79,80], which have been useful for documenting tissue-specific patterns of gene expression and widely used to construct coexpression networks [81,82]. However, there are not enough datasets that have provided comparable developmental profiles for buckwheat. The transcriptome dataset described here provides many insights into the dynamic nature of gene expression variation in buckwheat. In addition, transcriptome analysis was conducted on two buckwheat organs with different biological characteristics, including leaves and roots of plants at the seeding stage. After high-throughput RNA-Seq, approximately trimmed high-quality reads mapped to unique positions in the gene set of the reference genome, with 19,092,767 (leaf) and 19,021,744 (root) predicted coding and non-coding gene models. A total of 3203 specific genes with organizational expression were identified. Functional annotation indicates that these genes are widely involved in the regulation of pathways such as phenylpropanoid biosynthesis, phase and glucose introversion, flatter biosynthesis, fatty acid degradation, and alpha linolenic acid metabolism. After IAA treatment, high tissue specificity was still maintained in roots and leaves, with a significant enrichment of major pathways including phenylpropanoid biosynthesis, phase and glucose inversion, flatter biosynthesis, fatty acid degradation, and alpha linolenic acid metabolism. At the same time, it was found that the differences in IAA response enhanced tissue differentiation, with a total of 3982 tissue-specific expressed genes identified. The significantly enriched pathways included plant hormone signal transduction and metabolic startup and crossover. In the translation of plant hormone signals, 11 genes responsible for responding to hormone signals were found to show changes in expression.

Based on the transcriptome analysis, the expression in the three organs exhibited a strong correlation with DEGs and metabolic pathways. These findings indicated that specific transcripts present in different tissues demonstrated significant tissue specificity in buckwheat at the seedling stage, despite the total number of genes expressed showing minimal variation across different tissues in the transcriptome.

## 5. Conclusions

Tartary buckwheat (*Fagopyrum tataricum*) is a type of cultivated medicinal and edible crop with good economic and nutritional value. High-throughput transcriptome analysis was used to analyze buckwheat leaf and root tissues before and after IAA treatment. During the three stages of leaf IAA treatment, a total of 739, 1232, and 1384 DEGs were identified. Among these stages, four pathways—plant hormone signal transduction, MAPK signaling pathway–plant, phenylpropanoid biosynthesis, and flavonoid biosynthesis—were consistently observed. In the roots of the three stages treated by IAA, 987, 475, and 2512 DEGs were detected, respectively. Four pathways—plant hormone signal transduction, MAPK signaling pathway–plant, and phenylpropanoid biosynthesis—were consistently obtained. Comparing the two tissues, the expression changes in response to IAA occurred earlier in the roots than in the leaves, with a large number of DEGs appearing in the early stages and rapidly decreasing in the later stages. However, the DEGs in the leaves increased with treatment time, aligning with the absorption and transportation characteristics of IAA in plants. A plant hormone signal transduction pathway significantly enriched in various organizational stages detected seven and 11 genes in the leaves and roots, respectively, participating in its regulation. Comparing the genes specifically expressed in leaves and roots, we found that the number of genes specifically expressed increased after IAA treatment. Specific enrichment was observed in different tissues in pathways such as plant hormone signal transduction and start-up and crossover metabolism. We also detected significant expression changes in 11 genes. This study utilized transcriptome analysis to investigate the expression regulation of various tissues of buckwheat in response to IAA hormone stimulation. This research provides a comprehensive dataset for understanding the regulation of buckwheat tissue development controlled by auxin.

## Figures and Tables

**Table 1:** Gene-specific primer for qRT-PCR analysis.

Gene ID	Sequence (5′-3′)
*FtPinG0000387700*	AGCCGAGGAGGCACTTACTA//GGCTAGAGATCACAGACGGC
*FtPinG0000683600*	AATGGTCTACCTGTGGCTGC//AATGCCTGAGGACTGACAGC
*FtPinG0000764000*	CTCACCACAGGTTGGAGCAT//GCTGCAAGGACACCAATGTG
*FtPinG0000807700*	AGCCGAAACGACACCTTCTT//GCTGCAAGGACACCAATGTG
*FtPinG0000840000*	TAGGCGAAGACCCACCCATA//GCCTGATCTTTCAGGGTGCT
*FtPinG0001741700*	GAGTGCTTGCAACTGCTTCC//CCGGTGCATCATCTCCTCAA
*FtPinG0001971700*	AATGCGGACCAAGCAAACAC//TTCACTGCGAAGCTCATGGT
*FtPinG0002469700*	GGAGTTGTGGCATGCTTGTG//TCATCAGTGTCTGGTTCCGC
*FtPinG0002499600*	CCTCAAAGATCAGGCTGGCA//GCCTTGTGGATCCATCTGCT
*FtPinG0002591700*	CCACAGGGACACATGGAACA//GACCTGCAACCTCCACATGA
*FtPinG0003227500*	CGCAGGCATTTGCTGACTAC//TTGGGTTGCTCTTCGGACTC
*FtPinG0003331700*	AAGCTGGCTCTGCTTCCATT//GCTCGTATCCGAAGCTGTCA
*FtPinG0004636500*	CCTCCTCAGCTGATTTGCCA//TAATTGGTCGGTTGCCTGCT
*FtPinG0005201700*	GGGCTGTTCAAAGCCAACAG//CCACCCGTTGAATCATCCCA
*FtPinG0005575800*	CTCGTCCAAGCATGGGTTCT//AAGGCGAACGGAAACCAGAT
*FtPinG0007231100*	TAATCGGCACAGCAGGGTTT//GTGACTCCCATTCCGCTTGA
*FtPinG0008443000*	GATACGAGTACTCACGGCGG//CCCGCGGTATATGTGACGAA
*FtPinG0008597700*	CGAAGATCCTCTGCCGTGTT//CAAAAAGAGTGCACGGTCGG
*FtPinG0009506900*	TCACCATGGGAATTGGAGCC//TGGAGGCGGAAAATGGTCTC

**Table 2:** Statistics of expressed genes through high-throughput transcriptome analysis.

Sample	Clean Reads Pairs	Clean Base (bp)	GC (%)	Q30 (%)	Total Read Pairs	Total Mapped Reads	Uniq Mapped Reads	Multiple Mapped Reads
Leaf-CK-1	20,728,710	6,218,613,000	46.32	89.90	20,728,710	19,559,891 (94.36%)	19,092,767 (92.11%)	467,124 (2.25%)
Leaf-CK-2	20,737,194	6,221,158,200	46.34	90.19	20,737,194	19,485,885 (93.97%)	19,021,744 (91.73%)	464,141 (2.24%)
Leaf-CK-3	20,117,528	6,035,258,400	46.09	90.46	20,117,528	18,874,534 (93.82%)	18,412,786 (91.53%)	461,748 (2.30%)
Leaf-1D-1	20,825,096	6,247,528,800	46.24	90.54	20,825,096	19,643,518 (94.33%)	19,162,072 (92.01%)	481,446 (2.31%)
Leaf-1D-2	21,184,145	6,355,243,500	46.17	89.97	21,184,145	19,936,268 (94.11%)	19,395,678 (91.56%)	540,590 (2.55%)
Leaf-1D-3	20,709,917	6,212,975,100	46.20	89.81	20,709,917	19,505,462 (94.18%)	18,893,395 (91.23%)	612,067 (2.96%)
Leaf-2D-1	20,764,665	6,229,399,500	46.49	90.09	20,764,665	19,408,654 (93.47%)	18,763,601 (90.36%)	645,053 (3.11%)
Leaf-2D-2	20,866,415	6,259,924,500	45.42	89.64	20,866,415	19,617,791 (94.02%)	19,170,153 (91.87%)	447,638 (2.15%)
Leaf-2D-3	21,089,542	6,326,862,600	46.06	90.26	21,089,542	19,691,229 (93.37%)	19,066,009 (90.41%)	625,220 (2.96%)
Leaf-3D-1	20,515,821	6,154,746,300	45.70	90.26	20,515,821	19,245,412 (93.81%)	18,798,736 (91.63%)	446,676 (2.18%)
Leaf-3D-2	20,706,805	6,212,041,500	46.03	90.19	20,706,805	19,426,273 (93.82%)	18,913,196 (91.34%)	513,077 (2.48%)
Leaf-3D-3	20,875,522	6,262,656,600	46.05	90.46	20,875,522	19,631,121 (94.04%)	18,858,644 (90.34%)	772,477 (3.70%)
Root-CK-1	20,444,463	6,168,821,000	44.48	90.12	20,444,463	19,145,496 (93.65%)	18,751,891 (91.72%)	393,605 (1.93%)
Root-CK-2	22,723,102	6,232,005,900	43.59	90.14	22,723,102	19,749,568 (86.91%)	19,288,155 (84.88%)	461,413 (2.03%)
Root-CK-3	22,117,521	6,361,707,400	44.49	90.08	22,117,521	19,376,695 (87.61%)	18,863,241 (85.29%)	513,454 (2.32%)
Root-1D-1	23,730,617	6,196,114,800	44.25	90.17	23,730,617	19,444,463 (81.93%)	18,938,103 (79.80%)	506,360 (2.13%)
Root-1D-2	19,731,775	6,374,886,000	44.10	90.15	19,731,775	19,723,102 (99.96%)	19,325,198 (97.94%)	397,904 (2.02%)
Root-1D-3	21,037,784	6,319,515,100	45.71	90.22	21,037,784	19,117,521 (90.87%)	18,603,542 (88.43%)	513,979 (2.44%)
Root-2D-1	23,131,324	6,220,229,600	45.27	90.22	23,131,324	19,730,617 (85.30%)	19,247,665 (83.21%)	482,952 (2.09%)
Root-2D-2	20,309,102	6,277,100,500	45.39	90.20	20,309,102	19,731,775 (97.16%)	19,243,672 (94.75%)	488,103 (2.40%)
Root-2D-3	20,975,996	6,206,517,800	46.16	90.23	20,975,996	19,037,784 (90.76%)	18,694,823 (89.12%)	342,961 (1.64%)
Root-3D-1	21,456,310	6,191,025,000	45.31	90.20	21,456,310	19,131,324 (89.16%)	18,612,160 (86.74%)	519,164 (2.42%)
Root-3D-2	20,059,779	6,098,242,500	45.24	90.15	20,059,779	19,309,102 (96.26%)	19,025,500 (94.84%)	283,602 (1.41%)
Root-3D-3	21,570,970	6,234,457,500	43.34	90.20	21,570,970	19,975,996 (92.61%)	19,747,583 (91.55%)	228,413 (1.06%)

Sample: Sample number; Clean read pairs: the number of double-ended reads in each sample after quality control; Clean base: the total number of bases measured in each sample after quality control; GC: the total number of G and C in clean bases constitutes the percentage of the total number of bases; Q30: the percentage of bases with mass number greater than 30. Total read pairs: the number of mapped sequences that have been mapped into a genome; Total mapped reads: data that are total mapped to the reference genome; Uniq mapped reads: data that are uniquely mapped to the reference genome; Multiple mapped reads: compared data that are mapped to Multiple locations in the reference genome.

## Data Availability

The data are contained within the article; further inquiries may be directed to the corresponding author.
